# Extension of the Lecture Term

**Published:** 1848-09

**Authors:** 


EXTENSION OF THE LECTURE TERM.

We have received the annual circulars of a considerable number of the Medical Colleges in the United States, from which, and the advertisements in the Medical Journals, we have gleaned the following information, by which their action in reference to an extension of the lecture term may be seen.

| Institution. | No. of Professors. | Length of Term. |
|---|---|---|
| Harvard, (Boston,) | 7 | Four months. |
| Berkshire, (Mass.) | 6 | Fourteen weeks. |
| Dartmouth, (N. H.) | 6 | Fourteen weeks. |
| Castleton, (Vermont,) | 6 | Four months. |
| Yale, (Conn.) | 6 | Sixteen weeks. |
| University of New York, | 6 | Four months. |
| Buffalo, " | 7 | Twenty weeks. |
| Geneva, " | 6 | Sixteen weeks. |
| Albany, " | 7 | Sixteen weeks. |
| Col. Phy. and Surg. " | 7 | Five months and a half. |
| University of Penn. | 7 | Five months and a half. |
| Jefferson Med. Col. | 7 | Four months and a half. |
| University of Maryland. | 6 | Four months and a half. |
| Hampden Sidney, Va. | 6 | Nearly five months. |
| University of Virginia. | 3 | Eight months. |
| Winchester Med. Col. | 5 | Seven months. |
| Med. Col. of S. C. | 7 | Five months. |
| Georgia Medical College. | 7 | Five months. |
| Memphis Med. Col. | 7 | Four months. |
| Transylvania Un., Ky. | 7 | Four months. |
| Un. of Louisville, " | 7 | Four months and a half. |
| St. Louis Univ. of Mo. | 8 | Four months and a half. |
| Univ. State of Missouri, | 6 | Four months and a half. |
| Ohio Med. Col. | 6 | Four months. |

The advertisement of Transylvania University states, that the "session will open on the first Monday in November next," and the Medical department of the University of the State of Missouri "on the 16th of October," but without mentioning when they will terminate; we infer, therefore, that the Lectures in these institutions will conclude about the first of March, as heretofore.

From the above table it will be seen that in one of the schools, the lecture term is eight months; in another, seven months; in two, five months and a half; in three, five months; in five, four months and a half; in six, four months; in three, sixteen weeks; in two, fourteen weeks.

It would appear that a majority of the schools, *including all of*

*those in the New England States*, and three out of five of the New York schools, continue to adhere to their former term. Much disparity likewise prevails in the number of lectures delivered during a course. The report of the committee on this subject, submitted to the convention which assembled in Philadelphia in 1847, argues that "It is next to an impossibility, that the strongest intellect can receive and well digest some half a dozen discourses or more a day, embracing subjects which have sometimes little or no immediate connection with each other." * * * * * "With a lengthened period for teaching, a double advantage will be gained; a wider extent of information may be imparted to the student, while his time will be occupied with *fewer lectures during the day.*"

Some of the schools which have most extended the period of their sessions, we understand have diminished the number of their daily lectures, so that there is not a corresponding increase in the amount of instruction given; while others, with a *less extension*, continue to give the same number of daily lectures, and thus add materially to the number of lectures, and consequently the amount of instruction imparted during the term.

The editor of the Annalist, one of the most able and zealous of the advocates of reform, referring to "the actions of the conventions on this subject," remarks: "We acquiesced, but very reluctantly, in the fixation of the term at five months, by the American Medical Association, and yet live in hopes that the future will see the period further increased."

Commenting on the annual circular of the University of New York, the editor observes: "The circular goes on to oppose, as if it were a *sine qua non*, the plan adopted in some institutions of reducing the number of daily lectures, while the terms of attendance are increased. With this plan we have no fellowship, and shall not stop to exhibit its demerits. *It is, it appears to us, no improvement on the old system.*

"We fully agree with the Faculty of the University, that six lectures a day, are not more than an industrious student can attend without injury to his health, or risk of becoming inattentive from fatigue. We did it during the three latter years of our studentship, taking notes of several of the lectures, attending to the hospital practice, and having besides, leisure of an evening to write out some of the notes taken during the day, and for dissecting during the session; and we were never better in health, nor happier, than when thus busily engaged."—*N. Y. Annalist, Aug*, 1*st.* 1848.

The Faculty of Jefferson Medical College, in their last *Annual*

*Announcement*, hold very nearly the same language : "The prevalent idea, that too much is attempted to be taught in the four months generally allotted to the medical session, is of more recent origin."

"The time usually employed in lectures during four days in the week is six hours, and it is acknowledged in all professions, and has been especially so by lawyers from Lord Coke downwards, that six hours daily ought to be devoted to professional reading. To lecture may be regarded as synonymous with 'to read;' consequently the medical student who listens to six lectures in the day may be looked upon as having been 'read to' for six hours; and there can be no essential difference between *reading* and being *read to*, except in the circumstance, that the latter is much easier for the student. The well informed and able lecturer adapts his elucidations more readily to the comprehension of his hearers than can be done in the best books. He has an opportunity of perceiving whether he is understood ; and should he think he is not, he modifies or repeats his instruction."

Our individual experience confirms these views. We have had a pretty extensive opportunity of judging of the operation of the system of full lectures and moderate sessions, and we are fully persuaded, that indolent young men with long purses, prefer long sessions and few lectures, while the more industrious, with moderate means and correct habits, are unwilling to lose an hour in the day, and prefer to learn as much as possible within the shortest practicable period.

